# Supplementary material for: Smoking changes adaptive immunity with persistent effects
Source: Nature. 2024 Feb 14;626(8000):827–35. doi: 10.1038/s41586-023-06968-8 (PMC10881394; doi:10.1038/s41586-023-06968-8)
Supplement: Supplementary file 1 — Graphical abstract [file 41586_2023_6968_MOESM1_ESM.pdf]

nature portfolio

https://doi.org/10.1038/s41586-023-06968-8

Supplementary information

Smoking changes adaptive immunity with persistent effects

In the format provided by the authors and unedited

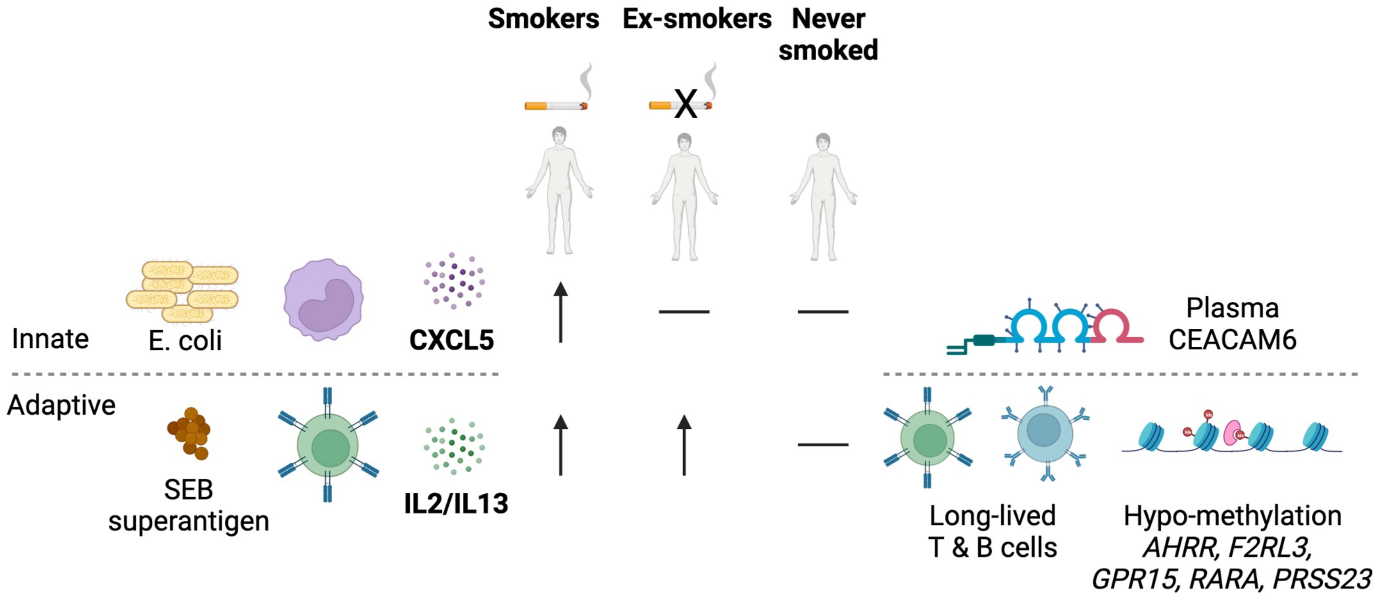

Supplementary Figure 1. Graphical abstract
Graphical abstract of the whole study showing groups of smokers, past-smokers and never-smokers and their associated features: CXCL5 is more induced in smokers compared to past-smokers and never-smokers upon innate stimulation, such as with E.coli, and this could be mediated through higher plasma levels of CEACAM6; whereas IL2 and IL13 are more induced in smokers and past-smokers compared to non-smokers upon adaptive immune stimulation, such as with SEB, and this could be mediated through memory T and B cells and hypo-methylation of signal transactivators and metabolism regulator genes (AHRR, F2RL3, GPR15, RARA, PRSS23).
