# Supplementary material for: Smoking changes adaptive immunity with persistent effects
Source: Nature. 2024 Feb 14;626(8000):827–35. doi: 10.1038/s41586-023-06968-8 (PMC10881394; doi:10.1038/s41586-023-06968-8)
Supplement: Supplementary file 2 — Reporting Summary [file 41586_2023_6968_MOESM2_ESM.pdf]

nature portfolio

Corresponding author(s): Violaine Saint-Andre & Darragh Duffy

Last updated by author(s): Oct 19, 2023

Reporting Summary

Nature Portfolio wishes to improve the reproducibility of the work that we publish. This form provides structure for consistency and transparency in reporting. For further information on Nature Portfolio policies, see our Editorial Policies and the Editorial Policy Checklist.

Statistics

For all statistical analyses, confirm that the following items are present in the figure legend, table legend, main text, or Methods section.

n/a	Confirmed	
☐	☒	The exact sample size (*n*) for each experimental group/condition, given as a discrete number and unit of measurement
☐	☒	A statement on whether measurements were taken from distinct samples or whether the same sample was measured repeatedly
☐	☒	The statistical test(s) used AND whether they are one- or two-sided<br>*Only common tests should be described solely by name; describe more complex techniques in the Methods section.*
☐	☒	A description of all covariates tested
☐	☒	A description of any assumptions or corrections, such as tests of normality and adjustment for multiple comparisons
☐	☒	A full description of the statistical parameters including central tendency (e.g. means) or other basic estimates (e.g. regression coefficient) AND variation (e.g. standard deviation) or associated estimates of uncertainty (e.g. confidence intervals)
☐	☒	For null hypothesis testing, the test statistic (e.g. *F*, *t*, *r*) with confidence intervals, effect sizes, degrees of freedom and *P* value noted<br>*Give P values as exact values whenever suitable.*
☒	☐	For Bayesian analysis, information on the choice of priors and Markov chain Monte Carlo settings
☒	☐	For hierarchical and complex designs, identification of the appropriate level for tests and full reporting of outcomes
☐	☒	Estimates of effect sizes (e.g. Cohen's *d*, Pearson's *r*), indicating how they were calculated

Our web collection on statistics for biologists contains articles on many of the points above.

Software and code

Policy information about availability of computer code

Data collection	Luminex xMAP technology software<br>FlowJo (Treestar)<br>Illumina Infinium MethylationEPIC BeadChip<br>Illumina HumanOmniExpress-24 and the HumanExome-12 BeadChips
Data analysis	R package relaimpo 2.2.3<br>R package ggplot2 3.2.1 (R 3.6.0)<br>MatrixEQTL 2.2 R package<br>heatmaply 1.0.0 and dendextend 1.13.12 with “complete” clustering method and “euclidien” distance in (R 3.6.0)<br>R 4.2.1 using FactoMineR 2.8

For manuscripts utilizing custom algorithms or software that are central to the research but not yet described in published literature, software must be made available to editors and reviewers. We strongly encourage code deposition in a community repository (e.g. GitHub). See the Nature Portfolio guidelines for submitting code & software for further information.

Data

Policy information about availability of data

All manuscripts must include a data availability statement. This statement should provide the following information, where applicable:

- Accession codes, unique identifiers, or web links for publicly available datasets
- A description of any restrictions on data availability
- For clinical datasets or third party data, please ensure that the statement adheres to our policy

SNP array data can be accessed from the European Genome-Phenome Archive EGA under accession EGAS00001002460, the Infinium MethylationEPIC raw intensity data can be accessed via the following link: https://dataset.owey.io/doi/10.48802/owey.f83a-1042, cytokine data is in Supplementary Data 1 and eCRF data is in Supplementary Data 2.

Human research participants

Policy information about studies involving human research participants and Sex and Gender in Research.

Reporting on sex and gender	The cohort was specifically designed to be equally balanced in terms of sex, 500 men and 500 women who were also stratified by age (20-69 years old). Sex (as opposed to gender) was confirmed by genetic chromosome analysis. For identification of environmental effects on cytokine responses we corrected for both age and sex. We also report specific sex effects on cytokine responses.
Population characteristics	The 1,000 donors of the Milieu Intérieur cohort were recruited by BioTrial (Rennes, France) to be composed of “healthy” individuals of the same genetic background (Western European) and to have 100 women and 100 men from each decade of life, between 20 and 69 years of age. Donors were selected based on various inclusion and exclusion criteria that were previously described (Thomas et al., 2015). Briefly, donors were required to have no history or evidence of severe/chronic/recurrent pathological conditions, neurological or psychiatric disorders, alcohol abuse, recent use of drugs, recent vaccine administration, and recent use of immune modulatory agents. To avoid the influence of hormonal fluctuations in women, pregnant and peri-menopausal women were not included. To avoid genetic stratification in the study population, the recruitment of donors was restricted to individuals whose parents and grandparents were born in Metropolitan France.
Recruitment	Human samples came from the Milieur Intérieur cohort, and were recruited by BioTrial (Rennes, France) during September 2011-July 2012. The study is sponsored by Institut Pasteur (Pasteur ID-RCB Number: 2012-A00238-35) and was conducted as a single center interventional study without an investigational product. The original protocol was registered under ClinicalTrials.gov (study# NCT01699893). The samples and data used in this study were formally established as the Milieu Intérieur biocollection (NCT03905993), with approvals by the Comité de Protection des Personnes – Sud Méditerranée and the Commission Nationale de l'Informatique et des Libertés (CNIL) on April 11, 2018. Donors were defined as healthy based on inclusion/exlcusion criteria previously described in Thomas et al, Clinical Immunology 2015.
Ethics oversight	The study was approved by Comité de Protection des Personnes – Ouest 6 on June 13th, 2012, and by the French Agence Nationale de Sécurité du Médicament (ANSM) on June 22nd, 2012.

Note that full information on the approval of the study protocol must also be provided in the manuscript.

Field-specific reporting

Please select the one below that is the best fit for your research. If you are not sure, read the appropriate sections before making your selection.

☒ Life sciences ☐ Behavioural & social sciences ☐ Ecological, evolutionary & environmental sciences

For a reference copy of the document with all sections, see nature.com/documents/nr-reporting-summary-flat.pdf

Life sciences study design

All studies must disclose on these points even when the disclosure is negative.

Sample size	The original sample size of the Milieu Interieur cohort (n=1000) was calculated to have sufficient power for strong to intermediate genetic effects. For this particular study no specific sample size was calculated and all available donors were included in each analysis step. Unless otherwise stated, all displayed results have been performed on the 956 individuals of the cohort who gave consent to share their data publicly in order to ensure easy reproducibility of the results
Data exclusions	For the CD3+CD28 stimulation, we identified through k-means clustering a group of 705 individuals that responded to the stimulation and a group of 295 individual did not. This lack of response of 295 individuals is explained by a FcγRIIA polymorphism (rs1801274) that was previously described as preventing response to this CD3+CD28 stimulation (Stein et al., Genes and Immunity, 2018) (Extended Data Fig. 9). All statistical analyses on CD3+CD28 stimulations in this study were thus performed on the 705 responders only.
Replication	Due to the specific nature of the cohort and associated data sets replication has not been performed.

Randomization	Samples were completely randomized prior to generation of cytokine data sets
Blinding	The generation of cytokine data sets was blinded as to the stimuli condition and donor ID

Reporting for specific materials, systems and methods

We require information from authors about some types of materials, experimental systems and methods used in many studies. Here, indicate whether each material, system or method listed is relevant to your study. If you are not sure if a list item applies to your research, read the appropriate section before selecting a response.

Materials & experimental systems

n/a	Involved in the study
☐	☒ Antibodies
☒	☐ Eukaryotic cell lines
☒	☐ Palaeontology and archaeology
☒	☐ Animals and other organisms
☐	☒ Clinical data
☒	☐ Dual use research of concern

Methods

n/a	Involved in the study
☒	☐ ChIP-seq
☒	☐ Flow cytometry
☒	☐ MRI-based neuroimaging

Antibodies

Antibodies used	Supernatants from TruCulture tubes were analyzed by Rules Based Medicine (Austin, Texas, US) using the Luminex xMAP technology. Samples were analyzed according to the Clinical Laboratory Improvement Amendments (CLIA) guidelines. The lower limit of quantification (LLOQ) was determined as previously described (Duffy et al., Immunity 2014), and is the lowest concentration of an analyte in a sample that can be reliably detected and at which the total error meets CLIA requirements for laboratory accuracy. Information on the antibody clones used in the assay is the propriety of Rules Based Medicine.
Validation	Validation of each antibody pair used in the Luminex assay has been performed by Rules Based Medicine in respect of CLIA guidlines and testing for specificity, sensitivity and cross-reactivity.

Clinical data

Policy information about clinical studies

All manuscripts should comply with the ICMJE guidelines for publication of clinical research and a completed CONSORT checklist must be included with all submissions.

Clinical trial registration	NCT01699893, NCT03905993
Study protocol	Clinical trials.gov : NCT01699893, NCT03905993
Data collection	Donors were recruited by BioTrial (Rennes, France) during September 2011-July 2012. Data has been generated from these samples since 2012.
Outcomes	NA
